# Antimicrobial actions of the human epididymis 2 (HE2) protein isoforms, HE2alpha, HE2beta1 and HE2beta2

**DOI:** 10.1186/1477-7827-2-61

**Published:** 2004-08-24

**Authors:** Suresh Yenugu, Katherine G Hamil, Frank S French, Susan H Hall

**Affiliations:** 1 Laboratories for Reproductive Biology, Department of Pediatrics, University of North Carolina, Chapel Hill, NC 27599-7500, USA

## Abstract

**Background:**

The HE2 gene encodes a group of isoforms with similarities to the antimicrobial beta-defensins. We demonstrated earlier that the antimicrobial activity of HE2 proteins and peptides is salt resistant and structure dependent and involves permeabilization of bacterial membranes. In this study, we further characterize the antimicrobial properties of HE2 peptides in terms of the structural changes induced in E. coli and the inhibition of macromolecular synthesis.

**Methods:**

E. coli treated with 50 micro g/ml of HE2alpha, HE2beta1 or HE2beta2 peptides for 30 and 60 min were visualized using transmission and scanning electron microscopy to investigate the impact of these peptides on bacterial internal and external structure. The effects of HE2alpha, HE2beta1 and HE2beta2 on E. coli macromolecular synthesis was assayed by incubating the bacteria with 2, 10 and 25 micro g/ml of the individual peptides for 0–60 min and measuring the incorporation of the radioactive precursors [methyl-^3^H]thymidine, [5-^3^H]uridine and L-[4,5-^3^H(N)]leucine into DNA, RNA and protein. Statistical analyses using Student's t-test were performed using Sigma Plot software. Values shown are Mean ± S.D.

**Results:**

E. coli treated with HE2alpha, HE2beta1 and HE2beta2 peptides as visualized by transmission electron microscopy showed extensive damage characterized by membrane blebbing, thickening of the membrane, highly granulated cytoplasm and appearance of vacuoles in contrast to the smooth and continuous membrane structure of the untreated bacteria. Similarly, bacteria observed by scanning electron microscopy after treating with HE2alpha, HE2beta1 or HE2beta2 peptides exhibited membrane blebbing and wrinkling, leakage of cellular contents, especially at the dividing septa, and external accumulation of fibrous materials. In addition, HE2alpha, HE2beta1 and HE2beta2 peptides inhibited E. coli DNA, RNA and protein synthesis.

**Conclusions:**

The morphological changes observed in E. coli treated with epididymal HE2 peptides provide further evidence for their membrane dependent mechanism of antibacterial action. HE2 C-terminal peptides can inhibit E. coli macromolecular synthesis, suggesting an additional mechanism of bacterial killing supplementary to membrane permeabilization.

## Introduction

Antimicrobial proteins and peptides are widely expressed in both plants and animals. A variety of natural antibiotics belonging to different classes such as defensins, cathelicidins, cercopins and protease inhibitors [1] are found in epithelial tissues of organs that are most likely exposed to pathogens. Among them, the most studied in humans are the defensins, which are broadly classified into three types viz alpha, beta and theta defensins depending on their disulfide bonding, tissue distribution and genomic organization. They exhibit broad spectrum antimicrobial activity [2-5], thus may form an important component of the innate immune system. Antimicrobial proteins and peptides including defensins are generally cationic in nature [6] and are believed to exert their bactericidal effect by permeabilizing the bacterial membranes by forming pores [7], thinning the membrane [8], or by destabilizing the membrane bilayer [9]. In addition to membrane permeabilization, antimicrobial proteins and peptides kill bacteria by inhibition of macromolecular biosynthesis [10-12] and/or interacting with specific vital components inside the bacteria [13,14].

**Figure 1:**
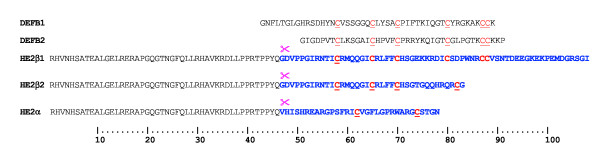
Amino acid sequence alignment of epididymal HE2 peptides with human β-defensins 1 and 2. Amino acid sequence shown in blue corresponds to the C-terminal peptides used in this study. The characteristic β-defensin six cysteine motif is represented in red.  represents the cleavage site where the full length proteins are cleaved to release the C-terminal peptides.

In the epididymis, a major organ of the male reproductive tract, immature sperm released from the testis undergo sequential maturation to acquire forward motility and fertilizing ability. A wide variety of proteins including antimicrobial proteins released into the lumen of epididymis bind sperm and are thought to play an important role in epididymal immunity in addition to their role in sperm maturation [15]. Examples of antimicrobial proteins reported in the male reproductive tract include human cationic antimicrobial protein (hCAP18, a cathelicidin) [16], defensins [17-20], the epididymal β-defensin member Bin1b [21], cystatins [22,23], lactoferrin [24] seminalplasmin [25] and seminogelin-derived peptides [26]. Earlier we identified and characterized the sperm binding epididymal proteins of the HE2 family [27], which show homology to the antimicrobial β-defensins. The HE2 gene located on chromosome 8p23 within the β-defensin gene cluster, encodes a series of isoforms containing identical proregions joined to different C-terminal peptides [27]. Among them, HE2β1 conserves the characteristic β-defensin-like six-cysteine motif (Figure 1). Furthermore, like the β-defensins, HE2 C-terminal peptides are cleaved from their proregions by a furin-like proprotein convertase and these peptides are reported to exist in the epididymal epithelium, luminal fluid and the seminal plasma [28]. We demonstrated the antimicrobial activity of HE2α, HE2β1 and HE2β2 proteins and their C-terminal peptides [29] and the epididymis specific defensin DEFB118 [30] against *E. coli*. Their antimicrobial activities are structure dependent and salt tolerant and their mechanism of action involves interacting with and permeabilizing bacterial membranes. However, structural evidence for the membrane changes in *E. coli *induced by these peptides is still lacking. Further, it is not still clear whether bacterial killing by HE2 peptides involves only membrane permeabilization or whether the peptides interact with specific targets inside the bacteria to inhibit metabolic processes as reported for other antimicrobial proteins is not yet demonstrated. In this study, using transmission and scanning electron microscopy, we provide further evidence that HE2 peptides induce significant structural changes in *E. coli *consistent with their membrane dependent mechanism of action as reported earlier. Further, we show that HE2 peptides inhibit *E. coli *DNA, RNA and protein synthesis suggesting that their antimicrobial action may also involve targets inside the bacteria as well as membrane permeabilization.

## Methods

### Recombinant peptide preparation and synthesis

HE2α and HE2β2 C-terminal peptides were synthesized at the Peptide Synthesis Facility, University of North Carolina, Chapel Hill by standard f-moc solid phase procedures using Rainin symphony multiple peptide synthesizer (Rainin Instrument, Woburn, MA). The purified peptides eluted as single peaks upon reverse phase high performance liquid chromatography (HPLC) and were further demonstrated to have their corresponding molecular weight by MALDI-TOF mass spectrometry. HE2β1 C-terminal peptide was expressed in *E. coli *and purified as described previously [29]. Briefly, *E. coli *strain M15 (pREP4) was transformed with pQE30 vector (Qiagen, Valencia, CA, U.S.A) containing cDNA that codes for HE2β1 C-terminal peptide. Protein expression was induced with 1 mM isopropyl-β-D-thiogalactoside for 1 h at 37°C and the His-tagged recombinant peptide was purified using nickel-nitrilotriacetate agarose column (Qiagen, Valencia, CA, U.S.A). To avoid baseline expression of the protein prior to induction, 1% glucose was maintained in the bacterial medium and the induction time was kept to a minimum (1 h) to minimize the toxic effects of the peptide on *E. coli*. The peptide was dialyzed extensively against 10 mM sodium phosphate (pH 7.4) to remove urea.

### Transmission electron microscopy

*E. coli *resuspended in 10 mM sodium phosphate buffer (pH 7.4) were treated with 50 μg/ml HE2α, HE2β1 or HE2β2 for 30 and 60 min. After incubation, bacterial cells were washed with 10 mM sodium phosphate buffer (pH 7.4) and fixed with an equal volume of 4% glutaraldehyde in 0.1 M sodium cacodylate buffer, pH 7.4, followed by centrifugation at 1000 rpm for 10 minutes to concentrate the cells in a pellet. The fixed samples were stored overnight to several days at 4°C in the fixative solution. The pellet was rinsed in 0.1 M sodium cacodylate buffer several times, and post-fixed with a combination of 1.25% potassium ferrocyanide and 1% buffered osmium tetroxide for one hour at room temperature. Following dehydration with a graded series of ethanols (30–100%) and two changes of propylene oxide, the cell pellet was infiltrated and embedded in PolyBed 812 epoxy resin (Polysciences, Inc., Warrington, PA). Ultra thin sections (70 nm) were cut and mounted on copper grids followed by post staining with 4% uranyl acetate and 0.4% lead citrate. The sections were examined and photographed at an accelerating voltage of 80 kV using a LEO EM 910 transmission electron microscope (LEO Electron Microscopy, Inc., Thornwood, NY) equipped with a Gatan BioScan digital camera (Gatan, Inc., Pleasanton, CA).

### Scanning electron microscopy

The structural changes induced by HE2 peptides on *E. coli *were studied using scanning electron microscopy as described earlier [30]. Bacterial cells suspended in 10 mM sodium phosphate buffer (pH 7.4) after treating with 50 μg/ml of HE2 peptide were fixed with an equal volume of 4% glutaraldehyde in 0.15 M sodium phosphate buffer, pH 7.4. Immediately following the addition of the fixative solution, the sample tube was mixed by gently inverting the tube up and down for several minutes to prevent clumping of the cells. The fixed samples were stored overnight to several days at 4°C in the fixative solution. Using a microanalysis vacuum filter holder (Fisher Scientific, Suwanee, GA) and a 0.1 μm polycarbonate membrane filter (Poretics Corporation, Livermore, CA), the suspended fixed cells were vacuum-filtered onto the membrane substrate, rinsed with 0.15 M sodium phosphate buffer, and dehydrated through a graded series of ethanols (30–100%). During the entire filtration, rinsing, and dehydration process, the cells were kept covered with fluid to prevent air drying. The filters were transferred in 100% ethanol to a critical point dryer (Balzers CPD-020, Bal-Tec AG, Vaduz, Liechtenstein), and dried using carbon dioxide as the transition solvent. The filters were mounted on aluminum specimen supports with carbon adhesive tabs, and coated with a 15 nm thickness of gold-palladium metal (60:40 alloy) using a Hummer X sputter coater (Anatech, Ltd., Alexandria, VA). Samples were examined with a Cambridge Stereoscan 200 scanning electron microscope (LEO Electron Microscopy, Inc., Thornwood, NY) using an accelerating voltage of 20 kV.

### Macromolecular synthesis

The effects of HE2 peptides on *E. coli *DNA, RNA and protein synthesis were studied as functions of incorporation of the radioactive precursors [methyl-^3^H]thymidine, [5-^3^H]uridine and L-[4,5-^3^H(N)]leucine respectively as described [30]. 1 × 10^6 ^mid-log phase *E. coli *resuspended in 10 mM sodium phosphate buffer (pH 7.4) were treated with varying concentrations of HE2 peptides and 2.5 μl/ml of either [methyl-^3^H]thymidine (20 Ci/mmol), [5-^3^H]uridine (25.5 Ci/mmol) or L-[4,5-^3^H(N)]leucine (59.5 Ci/mmol) for different time periods. After incubation, bacterial suspensions were added to 10% ice-cold trichloroacetic acid and allowed to stand in ice for 40 min. Samples were then collected on 2.4 cm GF/C glass microfiber filters (Fisher Scientific, Pittsburgh, PA) using vacuum filtration and washed thoroughly with 5% TCA and 70% ethanol. The filters were then dried and placed in scintillation vials containing 5 ml of EcoScint scintillation cocktail (National Diagnostics, Atlanta, GA) and counts were obtained in a LKB 1214 Rackbeta liquid scintillation counter (LKB WALLACE, Turku, Finland) for 1 min for each filter. Statistical analyses using Student's t-test were performed using Sigma Plot software (SPSS Inc., Chicago, IL). Values shown are Mean ± S.D.

## Results

### Transmission electron microscopy

**Figure 2:**
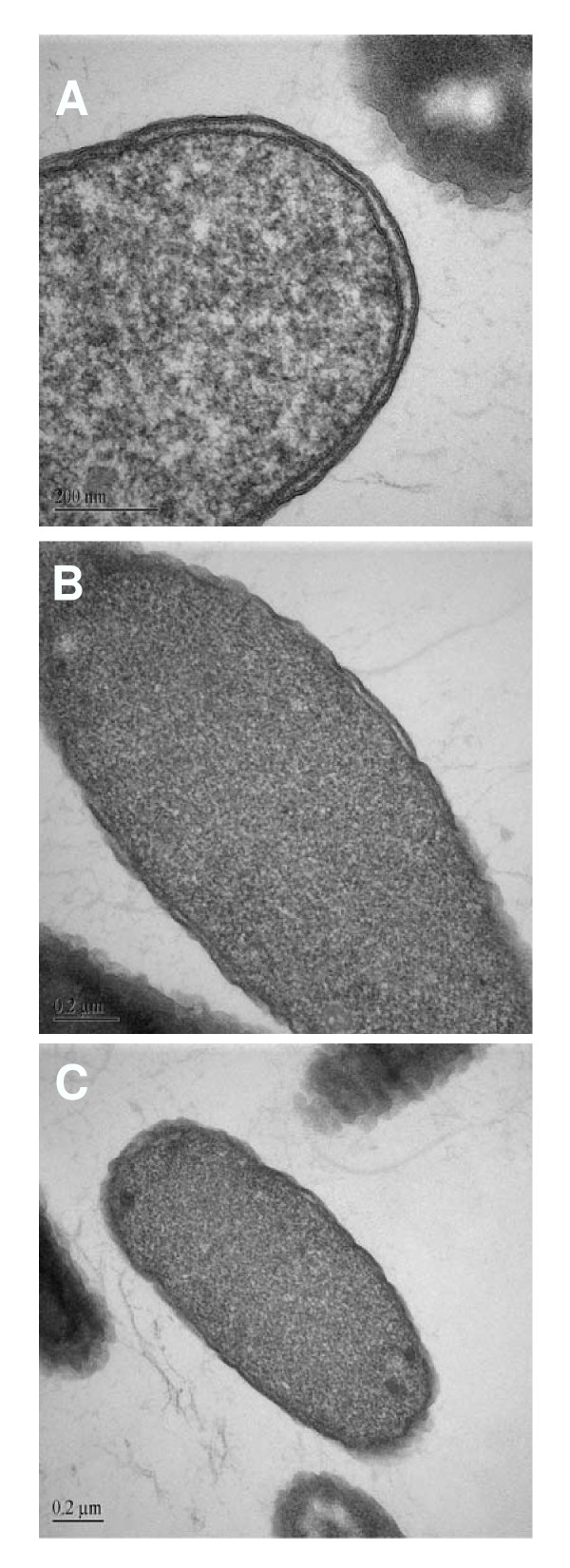
Transmission electron micrographs of untreated *E. coli *showing a smooth continuous double membrane structure.

**Figure 3:**
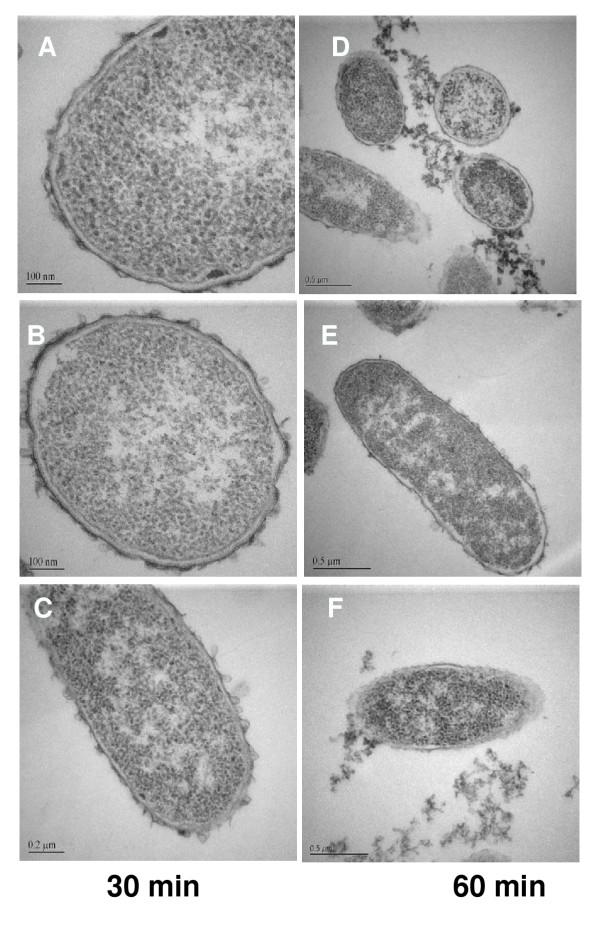
*E. coli *treated with 50 μg/ml HE2α peptide for 30 min (A-C) and 60 min (D-F) visualized by transmission electron microscopy showed membrane thickening and blebbing with subsequent leakage of cellular contents.

**Figure 4:**
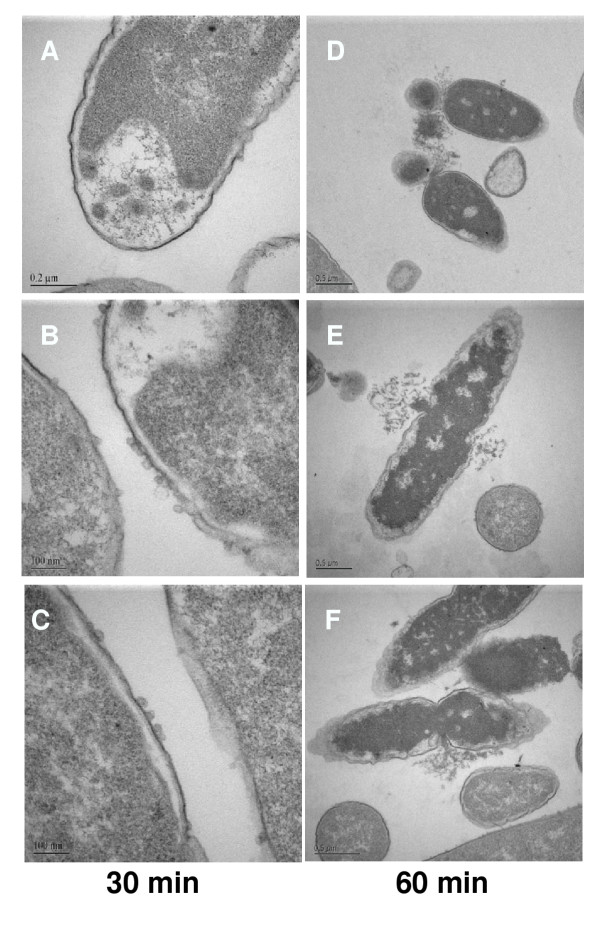
Transmission electron micrographs showing cytoplasmic retraction and extensive granulation of *E. coli *treated with 50 μg/ml HE2β1 peptide for 30 min (A-C) and 60 min (D-F).

**Figure 5:**
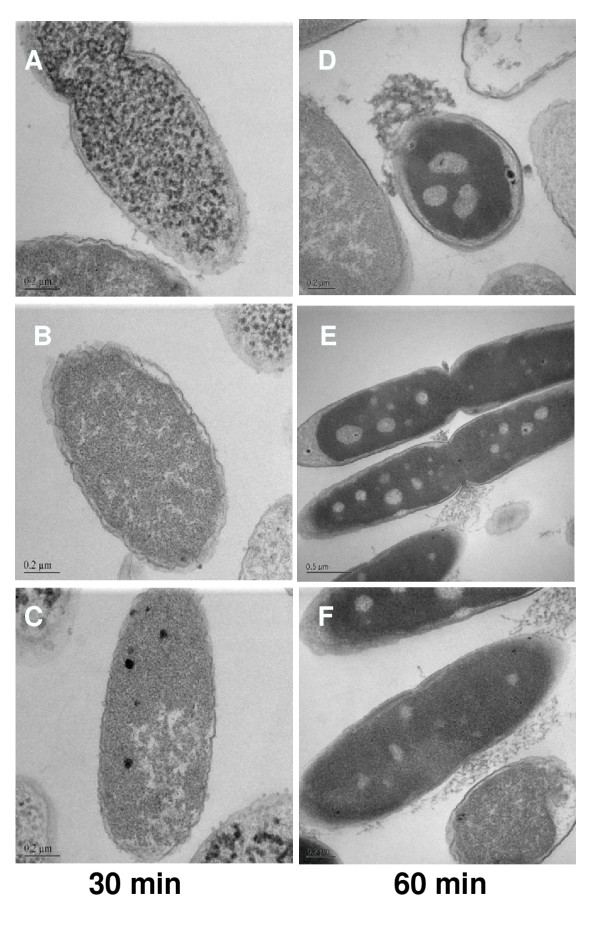
Incubation of *E. coli *with 50 μg/ml HE2β2 peptide for 30 min (A-C) and 60 min (D-F) show discontinuous membrane structure with extensive vacuole formation. Cellular contents appear to leak at the dividing septa.

Transmission electron microscopy revealed striking structural alterations in *E. coli *exposed to HE2 peptides. The three images shown for each treatment were documented in different fields of view and are intended to represent the range of responses seen in the bacteria. In contrast to the smooth continuous double membrane structure clearly visible in untreated bacteria (Fig. 2A,2B,2C), the outer membranes of bacteria treated with 50 μg/ml HE2α peptide for 30–60 min showed thickening and the protrusion of irregular blebs. The inner membrane was indistinct in many regions after 30 min and the cytoplasm was retracting from the outer membrane (Fig. 3A,3B,3C). After 60 min of HE2α treatment, the inner membrane was difficult to discern and fibrous and granular material, presumably cell contents appeared to exude from the damaged membranes (Fig. 3D,3E,3F). Treatment with 50 μg/ml HE2β1 peptide for 30 min resulted in numerous mushroom shaped blebs and retraction of cytoplasm (Fig. 4A,4B,4C) and by 60 min, these bacteria appeared to lose cell contents particularly at the division septa (Fig. 4D,4E,4F). Similarly, the HE2β2-treated bacteria showed loss of the double membrane structure, formation of blebs and outer membrane roughening (Fig. 5A,5B,5C). By 60 min, numerous large vacuoles accumulated, the cytoplasm was extensively granulated and retracted from the outer membrane and cell contents appeared to escape at division septa (Fig. 5D,5E,5F). The peptides appeared to induce structural changes specific to each peptide besides the morphological changes that were generally observed. HE2α peptide caused membrane thickenings, which was not observed with the other two peptides. Similarly, HE2β1 peptide caused retraction of cytoplasm when treated for 30 min, whereas HE2β2 peptide induced retraction of cytoplasm after 60 min incubation. Formation of vacuoles was more evident upon treating *E. coli *with HE2β2 peptide.

### Scanning electron microscopy

**Figure 6:**
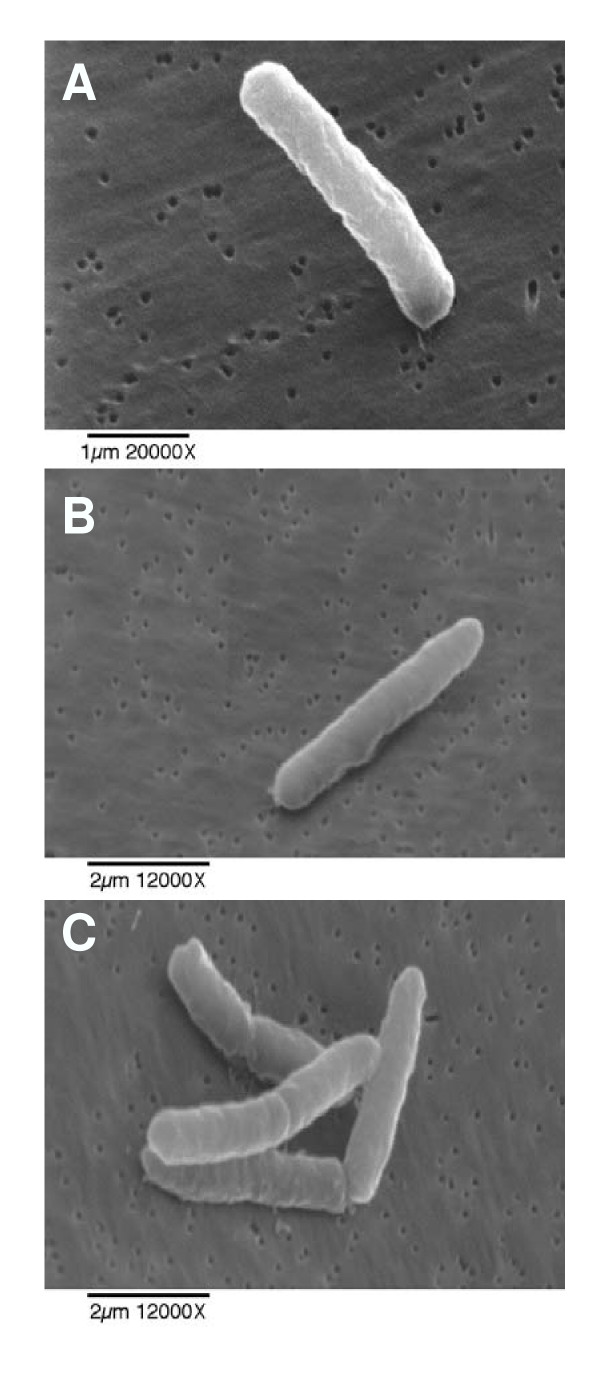
Scanning electron micrographs of untreated *E. coli *revealing a smooth membrane surface morphology.

**Figure 7:**
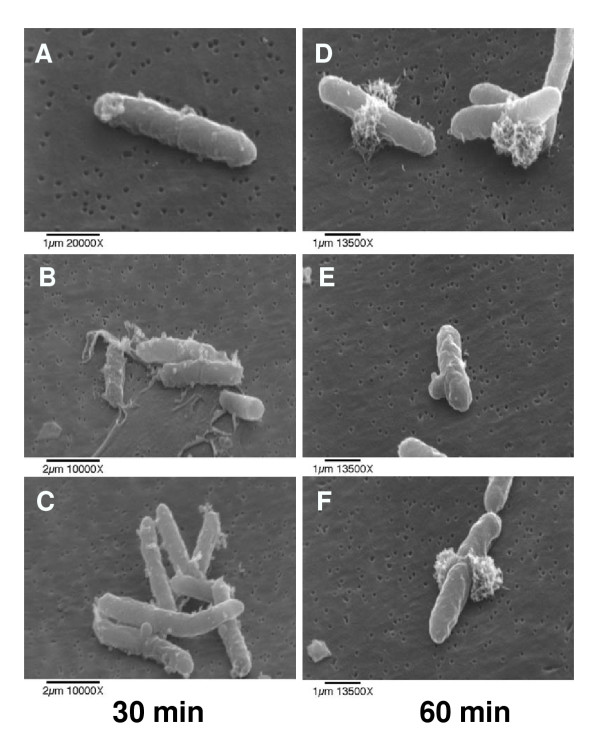
*E. coli *treated with 50 μg/ml HE2α peptide for 30 min (A-C) and 60 min (D-F) visualized by scanning electron microscopy show membrane blebbing and leakage of cellular contents.

**Figure 8:**
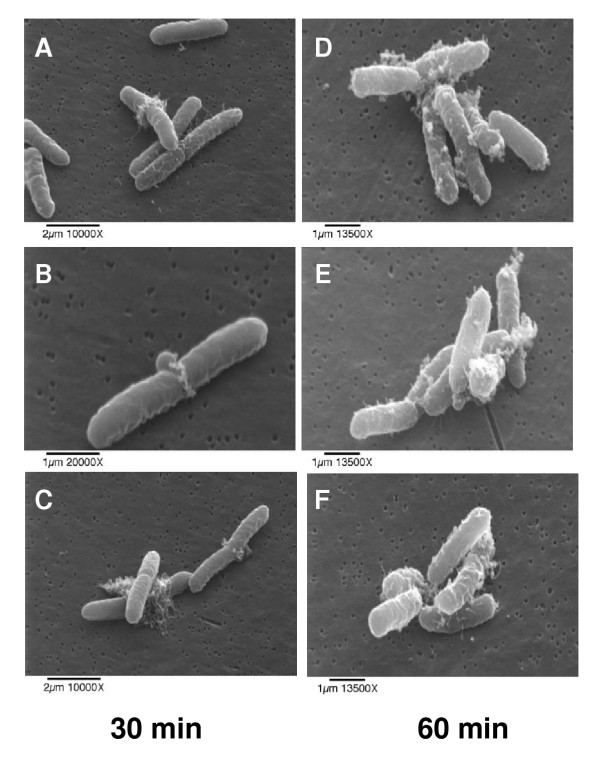
Membrane wrinkling and blebbing were evident in *E. coli *treated with 50 μg/ml HE2β1 peptide for 30 min (A-C) and 60 min (D-F).

**Figure 9:**
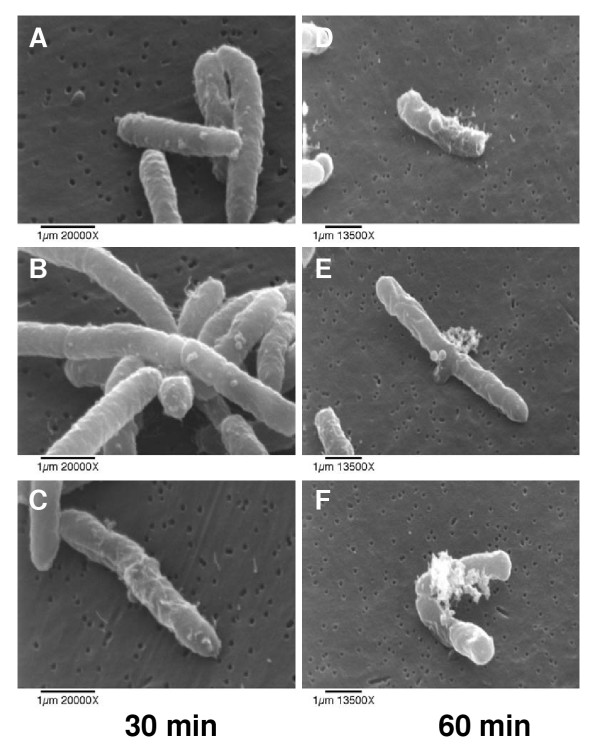
Scanning electron micrographs of *E. coli *treated with 50 μg/ml HE2β2 peptide for 30 min (A-C) and 60 min (D-F). Loss of bacterial membrane integrity due to surface blebbing and wrinkling was evident.

*E. coli *treated with HE2 peptides were observed using scanning electron microscopy to gain further insights into the membrane effects. The three images shown for each treatment were documented in different fields of view and are intended to represent the range of responses seen in the bacteria. Untreated bacterial cells had normal and smooth surface morphology (Fig. 6A,6B,6C). Bacteria treated with HE2α (Fig. 7A,7B,7C,7D,7E,7F), HE2β1 (Fig. 8A,8B,8C,8D,8E,8F) or HE2β2 (Fig. 9A,9B,9C,9D,9E,9F) peptides showed pronounced changes in their morphology consistent with the changes observed using transmission electron microscopy. *E. coli *treated with the HE2 peptides for 30–60 min showed pronounced wrinkling, surface roughening and blebbing of the membrane. A majority of the cells appeared to have lost their bacterial membrane integrity. The fibrous material and cellular debris, possibly arising due to leakage and cell lysis accumulated particularly at the dividing septa.

### Macromolecular synthesis

**Figure 10:**
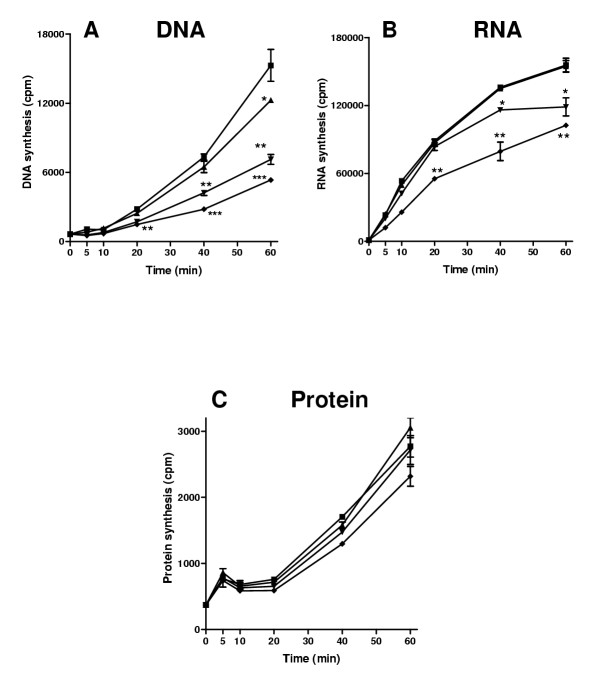
Effect of HE2α peptide on macromolecular synthesis in *E. coli*. A, [methyl-^3^H]thymidine incorporation into DNA. B, [5-^3^H]uridine incorporation into RNA. C, L-[4,5-^3^H(N)]leucine incorporation into proteins. 0 μg/ml (■); 2 μg/ml (▲); 10 μg/ml (▼); 25 μg/ml (◆). Values shown are mean ± SD. *, P < 0.05-0.01, **, P < 0.01-0.001, ***, P < 0.001 compared to 0 μg/ml at the corresponding time point.

To investigate whether HE2 peptides affect macromolecular synthesis of *E. coli*, the incorporation of radioactive precursors viz [methyl-^3^H]thymidine, [5-^3^H]uridine and L-[4,5-^3^H(N)]leucine into DNA, RNA and protein was studied in the presence of 2–25 μg/ml peptides. A dose and time dependent inhibition of DNA synthesis by HE2α peptide was observed (Fig. 10A). 2 μg/ml HE2α peptide inhibited DNA synthesis after 60 min incubation, whereas 10 and 25 μg/ml significantly inhibited DNA synthesis after 20 min incubation (Fig. 10A). RNA synthesis was not inhibited by 2 μg/ml HE2α peptide, whereas inhibition was observed with 10 and 25 μg/ml concentrations (Fig. 10B). No significant inhibition of protein synthesis by HE2α peptide was observed at any of the concentrations tested (Fig. 10C).

**Figure 11:**
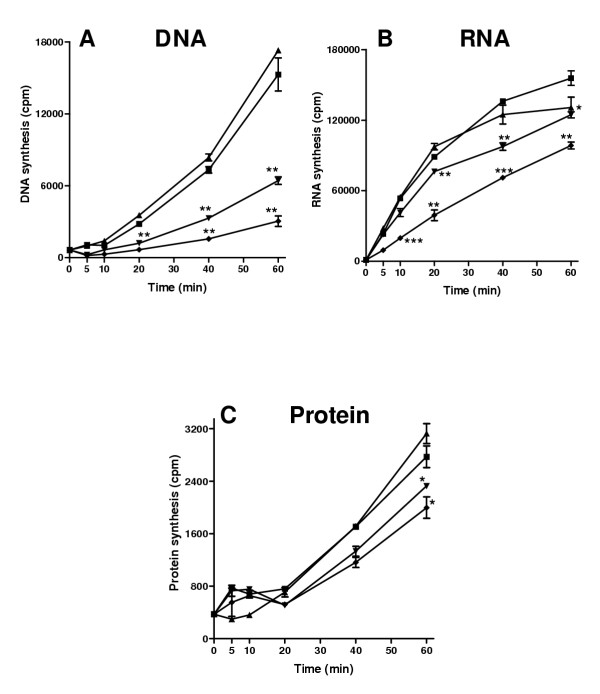
Effect of HE2β1 peptide on macromolecular synthesis in *E. coli*. A, [methyl-^3^H]thymidine incorporation into DNA. B, [5-^3^H]uridine incorporation into RNA. C, L-[4,5-^3^H(N)]leucine incorporation into proteins. 0 μg/ml (■); 2 μg/ml (▲); 10 μg/ml (▼); 25 μg/ml (◆). Values shown are mean ± SD. *, P < 0.05-0.01, **, P < 0.01-0.001, ***, P < 0.001 compared to 0 μg/ml at the corresponding time point.

In the case of HE2β1 peptide, 2 μg/ml dose did not inhibit DNA synthesis, whereas 10 and 25 μg/ml concentrations showed significant inhibition (Fig. 11A) after a 20 min incubation. Similarly, significant inhibition of RNA synthesis was not observed with 2 μg/ml. However, 10 and 25 μg/ml concentrations inhibited RNA after 20 min (Fig. 11B) and protein synthesis (Fig. 11C) after a 60 min incubation.

**Figure 12:**
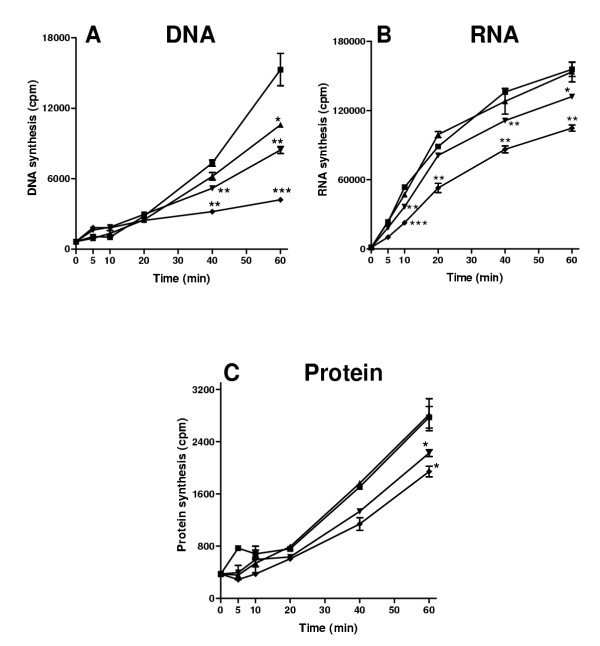
Effect of HE2β2 peptide on macromolecular synthesis in *E. coli*. A, [methyl-^3^H]thymidine incorporation into DNA. B, [5-^3^H]uridine incorporation into RNA. C, L-[4,5-^3^H(N)]leucine incorporation into proteins. 0 μg/ml (■); 2 μg/ml (▲); 10 μg/ml (▼); 25 μg/ml (◆). Values shown are mean ± SD. *, P < 0.05-0.01, **, P < 0.01-0.001, ***, P < 0.001 compared to 0 μg/ml at the corresponding time point.

Inhibition of *E. coli *DNA synthesis by HE2β2 peptide was dose and time dependent. Significant inhibition of DNA synthesis was observed after a 60 min incubation with 2 μg/ml HE2β2 peptide, whereas the inhibition was observed at a earlier time point with 10 and 25 μg/ml concentrations (Fig. 12A). However, RNA synthesis was not inhibited by 2 μg/ml HE2β2 peptide, whereas 10 and 25 μg/ml doses were effective after 40 and 10 min incubations respectively (Fig. 12B). Protein synthesis was inhibited only with 10 and 25 μg/ml HE2β2 peptide after a 60 min incubation (Fig. 12C). It appears that HE2 peptides inhibit DNA synthesis to a greater extent than RNA and protein synthesis, suggesting that DNA synthesis may be the sensitive target for antimicrobial action after membrane permeabilization.

## Discussion

Earlier we demonstrated that HE2 proteins and their C-terminal peptides exhibit salt tolerant and structural dependent antimicrobial activities and their mechanism involved permeabilization of both outer and inner bacterial membranes [29]. In this study, structural changes induced in *E. coli *by epididymal HE2α, HE2β1 and HE2β2 peptides as visualized by transmission and scanning electron microscopy provide further evidence of the membrane dependent mechanism of bacterial killing. Such structural changes induced in *E. col*i by other antimicrobial proteins and peptides were reported previously. Membrane thickening as shown in Fig 3A,3B,3C,3D,3E,3F was reported in *E. coli *treated with human neutrophil peptides 1 and 2 (defensins) [14]. Similarly, retraction of cytoplasm and the appearance of vacuoles as shown in Fig 4A,4B,4C,4D,4E,4F were reported for *E. coli *treated with synthetic peptides of the antimicrobial protein apolipoprotein A-II [31]. Highly granular cytoplasm with discontinuous membrane was reported for *E. coli *treated with the antimicrobial peptide tigerinin-1 [32] similar to the changes shown in Fig. 5A,5B,5C,5D,5E,5F. Scanning electron micrographs of *E. coli *treated with HE2 peptides also revealed striking structural changes in their morphology. HE2 peptides caused membrane wrinkling, blebbing and leakage of fibrous material primarily at the dividing septa in *E. coli*. Such structural changes shown in Fig. 7,8,9 were earlier reported for other antimicrobial proteins viz the cathelicidin-derived peptide SMAP-29 [33], temporin-L [34], salmon antimicrobial protein [35] and the epididymal proteins ESC42 (DEFB118) [30] and EPPIN [36]. An interesting observation in this study is the leakage of fibrous material primarily at the dividing septa. It is known that cell division in *E. coli *involves annular constriction of all layers of the cell envelope and synthesis and assembly of new septal materials [37]. It is possible that during this dynamic remodeling process, the region of division septum formation to be particularly vulnerable to attack by antibacterial proteins.

The mechanism of action of antimicrobial proteins is primarily thought to be membrane dependent involving membrane permeabilization and disruption. Structural characteristics of antimicrobial peptides tend to play an important role in their mechanism of action. For example, β-defensins are cationic in nature and with β-sheet rich amphipathic structures stabilized by the three disulfide motif [38]. The cationic nature of β-defensins favors them to bind to and disrupt target membranes that are rich in anionic phospholipids. Similarly, HE2α, HE2β1 and HE2β2 peptides are cationic in nature with basic pIs. Our three dimensional structural analysis of HE2β1 peptide revealed that it is rich in β-sheet structure and its tertiary structure presents regional concentrations of basic and hydrophobic amino acids similar to β-defensins [29]. Such structural characteristics of HE2 peptides which resemble to those of β-defensins suggest that they bind to and disrupt the anionic target membranes and mediate bacterial killing similar to β-defensins. However, alternate mechanisms of antimicrobial action such as inhibition of macromolecular synthesis [10-12] and interaction with specific targets inside the bacterial cells [13,14] are proposed. HE2 peptides at 10 and 25 μg/ml concentrations inhibited DNA, RNA and protein synthesis suggesting that their antimicrobial action may include interference with metabolic functions of *E. coli*. Inhibition of macromolecular synthesis was reported for bactenectins [39], human neutrophil peptide-1 [40], pleurocidin derived peptides [41] and the epididymal defensin DEFB118 [30]. In this study, it appears that HE2 peptides were more effective in inhibiting the incorporation of [methyl-^3^H]thymidine than [5-^3^H]uridine and L-[4,5-^3^H(N)]leucine, suggesting DNA synthesis is more sensitive to their antimicrobial action. It is possible that in bacteria that are extensively damaged by HE2 peptides, inhibition of macromolecular synthesis may result simply from the total breakdown of the cells. However the electron micrographs show that only some bacteria appear to be exuding cell contents after the 30 minute treatment. Thus during the first 10–20 minutes exposure to HE2 peptides, some peptides may be entering through pores too small for major cytoplasmic release. The early inhibition of DNA and RNA synthesis in bacteria where little loss of cell contents has occurred, may result from specific interaction of the synthetic machinery with HE2 peptides. Further studies are required to identify specific molecular targets within the bacteria and to establish whether HE2 interactions with these targets can be beneficial to the host by slowing bacterial proliferation.

Increasing recognition of the ability of a number of proteins on the sperm surface to kill bacteria has led to the proposal that they may defend against microbial attack in both the male and female reproductive tracts. The cathelicidin hCAP18 on sperm is processed by the prostate-derived protease, gastricsin to release the active peptide ALL-38 and is found in the female reproductive tract after intercourse [42]. A member of the β-defensin family, DEFB126 also appears to have a role in fertility as a capacitation factor on sperm [43]. Similarly, the rat epididymis specific β-defensin Bin1b, appears to play an important role in sperm maturation [44]. Thus, these defense proteins may enhance the probability of successful fertilization in addition to helping prevent the spread of sexually transmitted diseases.

## Conclusions

In conclusion, we report that the epididymal antimicrobial peptides HE2α, HE2β1 and HE2β2 induce striking morphological changes in *E. coli *consistent with their membrane dependent mechanism of action [29]. In addition to membrane permeabilization, their antimicrobial mechanism involves inhibition of *E. coli *DNA, RNA and protein synthesis.

## Author's contributions

SY performed the electron microscopy studies, radioactive incorporation assays and wrote majority of the manuscript. KGH prepared the recombinant peptides. SHH and FSF supervised and coordinated the work and the preparation of the manuscript. All authors read, commented upon and approved the final manuscript.
